# A randomized study of telephonic care support in populations at risk for musculoskeletal preference-sensitive surgeries

**DOI:** 10.1186/1472-6947-13-21

**Published:** 2013-02-07

**Authors:** David R Veroff, Tamara Ochoa-Arvelo, Benjamin Venator

**Affiliations:** 1 Health Dialog, 60 State Street, Boston, MA, USA; 2 Blue Cross Blue Shield of Massachusetts, 401 Park Drive, Boston, MA, USA; 3 Independent Evaluation Researcher, 7 Kearsarge Street, Concord, NH, USA

## Abstract

**Background:**

The rate of elective surgeries varies dramatically by geography in the United States. For many of these surgeries, there is not clear evidence of their relative merits over alternate treatment choices and there are significant tradeoffs in short- and long-term risks and benefits of selecting one treatment option over another. Conditions and symptoms for which there is this lack of a single clear evidence-based treatment choice present great opportunities for patient and provider collaboration on decision making; back pain and joint osteoarthritis are two such ailments. A number of decision aids are in active use to encourage this shared decision-making process. Decision aids have been assessed in formal studies that demonstrate increases in patient knowledge, increases in patient-provider engagement, and reduction in surgery rates. These studies have not widely demonstrated the added benefit of health coaching in support of shared decision making nor have they commonly provided strong evidence of cost reductions. In order to add to this evidence base, we undertook a comparative study testing the relative impact on health utilization and costs of active outreach through interactive voice response technology to encourage health coaching in support of shared decision making in comparison to mailed outreach or no outreach. This study focused on individuals with back pain or joint pain.

**Methods:**

We conducted four waves of stratified randomized comparisons for individuals with risk for back, hip, or knee surgery who did not have claims-based evidence of one or more of five chronic conditions and were eligible for population care management services within three large regional health plans in the United States. An interactive voice response (IVR) form of outreach that included the capability for individuals to directly connect with health coaches telephonically, known as AutoDialog®, was compared to a control (mailed outreach or natural levels of inbound calling depending on the study wave). In total, the study include 24,167 adults with commercial and Medicare Advantage private coverage at three health plans and at risk for lumbar back surgery, hip repair/replacement, or knee repair/replacement.

**Results:**

Interactive voice response outreach led to 10.7 (P-value < .0001) times as many inbound calls within 30 days as the control. Over 180 days, the IVR group (“intervention”) had 67 percent (P-value < .0001) more health coach communications and agreed to be sent 3.2 (P-value < .0001) time as many DVD- and/or booklet-based decision aids. Targeted surgeries were reduced by 6.7 percent (P-value = .6039). Overall costs were lower by 4.9 percent (P-value = .055). Costs that were not related to maternity, cancer, trauma and substance abuse (“actionable costs”) were reduced by 6.5 percent (P-value = .0286).

**Conclusions:**

IVR with a transfer-to-health coach-option significantly increased levels of health coaching compared to mailed or no outreach and lead to significantly reduced actionable medical costs. Providing high levels of health coaching to individuals with these types of risks appears to have produced important levels of actionable medical cost reductions. We believe this impact resulted from more informed and engaged health care decision making.

## Background

Knee repair and replacement, hip repair and replacement, and lumbar back surgery are common and expensive treatments for patients with hip and knee osteoarthritis and herniated discs. The rates of these treatments vary dramatically in different geographies; this variation cannot be explained by diagnostic characteristics or preferences of the patients [1-3]. These conditions each have a range of treatment options that carry different near-term and longer-term risks and benefits; decisions about these treatments ideally should be made in concordance with patients’ well-informed values and preferences. This set of conditions, along with other conditions, is frequently referred to as preference-sensitive conditions.

Shared Decision Making is a process that intends to ensure treatment options are selected in accord with patient preferences and needs by engaging patients and clinicians in discussion to review best medical evidence together, to explore patients’ values, interests, and preferences, to help patients understand the implications of treatment choices on their needs and expectations, and ultimately to reach a collaborative decision about treatment [4] Tools and strategies to support shared decision making include patient decision aids, coaching programs intended to support decisions, and training programs for clinicians [5]. The premise behind Shared Decision Making is that supporting patients in becoming more informed participants in the decision making process is both the right thing to do and represents a major opportunity to address rising costs [6,7]. Many of these strategies have been well-documented to be effective at improving decisions. A Cochrane review of randomized control trials found evidence that shared decision making interventions (through “decision aids”) improved a variety of measures associated with decision knowledge and decision quality and tended to result in more conservative treatment options selected rather than invasive surgery options [8]. Evidence demonstrating the impact of in-person educational meetings is more limited [9].

Deploying Shared Decision Making on a broad scale holds significant policy and business interest. There is a growing call on the legislative, regulatory, and policy front for more active use of Shared Decision Making [10-14]. The potential of more active Shared Decision Making to result in lowered medical costs at the same time as “doing the right thing” for patients is also appealing to private purchasers of services [15].

Population-based shared decision making programs, driven by claims-based predictive models and utilizing broad-based outreach efforts to engage individuals at the point of their decisions, have been in active use for over 12 years [15]. An enduring challenge of these programs is to design outreach that encourages the right individuals—those with true need for support on decisions—to utilize Shared Decision Making services and to do so cost-effectively. A common approach to managing these two needs is to mail outreach to individuals in hopes that individuals with needs will read the mailed pieces and make effort to engage with the Shared Decision Making services. Unfortunately, this approach has quite low impact. In Health Dialog’s experience, only 1 to 3% of mailed individuals respond to mailed outreach by phoning a toll-free number indicated in the mailer and speaking with a health professional trained in Shared Decision Making.

In an effort to assess more effective approaches to spurring use of Shared Decision Making, we decided to test a natural voice interactive voice recognition (IVR) application with the capability to transfer participants from the application to a health coach (known as AutoDialog®). We designed a series of quality improvement studies to assess whether this new technology could be effective in driving use of Shared Decision Making services (in this case, as measured by increase in rates of health coaching interactions).

The objective of this paper is to consider the relative impact of targeting by an IVR call versus a standard mailer or no materials on health coach interaction rates, as well as health care costs and utilization for individuals at risk for musculoskeletal preference-sensitive surgeries.

## Methods

### Study population

The study population is comprised of individuals from three different health plan clients of Health Dialog identified from November 2004 to May 2005.

Individuals who at the time of the study were at least 18 years of age, had coverage from one of the participating health plans, were eligible to participate in Health Dialog’s care management program, and had a valid telephone number had potential to be selected for the study. Individuals without any of five chronic conditions (asthma, coronary heart disease, heart failure, chronic obstructive pulmonary disease, or diabetes) who were identified as being at elevated risk for preference-sensitive back, hip, or knee surgery by Health Dialog proprietary models were eligible for the study. Individuals who had no program eligibility during the measurement period or had claims evidence of one of the targeted surgeries prior to the start of the study were excluded from the analysis (Table 1).

**Table 1:** Study design characteristics

	**Intervention arm N (%)**	**Control arm N (%)**
Randomized	14,443	10,706
Excluded because had surgery prior to study start	100 (0.69%)	73 (0.68%)
Excluded because lost health plan eligibility prior to the start of the study	442 (3.06%)	367 (3.43%)
Included for all analyses	13,901	10,266

The study was designed as a quality improvement assessment with the purpose of improving the operations of care management services Health Dialog provided to our clients under a business associates agreement. All services provided and all data were managed to be HIPAA compliant. Because these studies were quality improvement assessments, there was not need or expectation for external institutional review board approval [16]. It is worth noting that the services tested in this case were strictly outreach activities in comparison to existing outreach services—potential harm from adding this outreach was extremely unlikely.

### Study design

The study was designed to enable the detection of differences in health coach communication levels between intervention and control groups. The study was conducted in four independent waves that differed in the following ways: the targeted conditions (in one wave only individuals with risk for lumbar back surgery were targeted, in all other waves, individuals with risks for hip and knee replacement surgery were also included), the health plan product types included (in one wave, only non-Medicare Advantage members were included; in all other waves, Medicare Advantage members were also included), the control group treatment (in one wave, the control group did not receive any outreach; in all other waves, the control group received a mailed postcard), the selection criteria for inclusion in the study (in one wave, a clinical marker model was used to define the targeting criteria; in all other waves, Health Dialog’s proprietary surgery prediction models were used), the randomization strata, and the allocation ratios between intervention and control. The variability in approach resulted from the business priorities for each of the participating health plans. Each wave was independently powered to assess the primary outcome variable, health coach communication levels. There was great interest and value in combining these independent waves in order to also assess impact on health care costs and utilization which were much less well-powered in the individual waves.

The study was conducted in four waves; two of the waves were with one health plan in different regional areas. Within each wave of the study, subjects were assigned to intervention (AutoDialog® IVR) or control study arms utilizing a stratified random sampling approach. The variables used as stratification variables differed by wave and included targeted condition (back, knee, or hip), product type (Medicare or non-Medicare), gender, age cohort (18–35, 36–54, 55–64, or 65+), and a cluster variable based on predicted future costs using a proprietary risk scoring algorithm from Health Dialog and prior year health care utilization rates. The allocation ratios differed by wave depending on the business needs and expectations of each of the participating health plans. See Table 2 for information about the allocation ratios and stratification variables by wave.

**Table 2:** Randomization description

**Wave**	**Start month**	**Allocation ratio**	**Stratifying variables**
Wave 1	Nov-04	3:1	Age category and gender
Wave 2	Dec-04	45:55	Age category, gender, and Medicare Advantage coverage flag
Wave 3	May-05	3:1	Gender, Medicare Advantage coverage flag and claims-based cluster variable*
Wave 4	May-05	1:1	Gender and claims-based cluster variable*

*Developed with SAS fastclus procedure using predicted costs, inpatient admissions, ED and specialist visits in prior 12 months at time of randomization.

We selected individuals for participation by applying uniform selection algorithms within each wave of the study. In one wave, the defining person-level characteristics for inclusion in the study were claims indicators that they were likely to be considering back surgery. In all other waves, we applied Health Dialog’s proprietary claims-based predictive models to separately assess the future risk of lumbar back surgery, hip replacement surgery, and knee replacement surgery. Risk threshold cut-points were tailored for each wave depending on the business priorities for the health plan partners (such as the relative priorities of utilizing health coaching resources to engage members with chronic conditions and preference sensitive conditions). Individuals with claims evidence of asthma, coronary heart disease, chronic obstructive pulmonary disease, diabetes, and heart failure were not included in the study. We applied systematic rules to all participants so as to eliminate individuals with recent prior outreach and/or contact with a health coach. Individuals who had, as a result of prior contact, asked not to be contacted were also eliminated from the study.

We targeted subjects in the intervention group by placing outbound IVR calls to them. The call scripts also enabled direct connection to a health coach. Subjects in the control groups received standard mail materials in three of the waves and no materials in one of the waves. The start date for analysis of each wave corresponds to the earliest date of the intervention or control treatment for any subject within that wave.

As a new approach to outreach, there were very high levels of operational attention to these campaigns. This study was in effect single-blinded, as health coaches and other Health Dialog staff were aware of the pilot. Health coaches were not instructed to treat control and intervention subjects differently, but they were aware that they were participating in a controlled study and had potential visibility into what group each individual was assigned.

### Intervention

We placed up to three outbound IVR call attempts to each targeted individual in the intervention group. When these calls connected with a live person, the system confirmed that the targeted individual was the person on the phone and then worked through a script as described below. When the calls connected with an answering machine, we left messages that were HIPAA-compliant and included a toll-free number to dial into the IVR system. Individuals dialing into IVR system line were offered the same questions as individuals directly reached by the application. Individuals who did not select a transfer received no other outreach specific to these studies.

**Figure 1:**
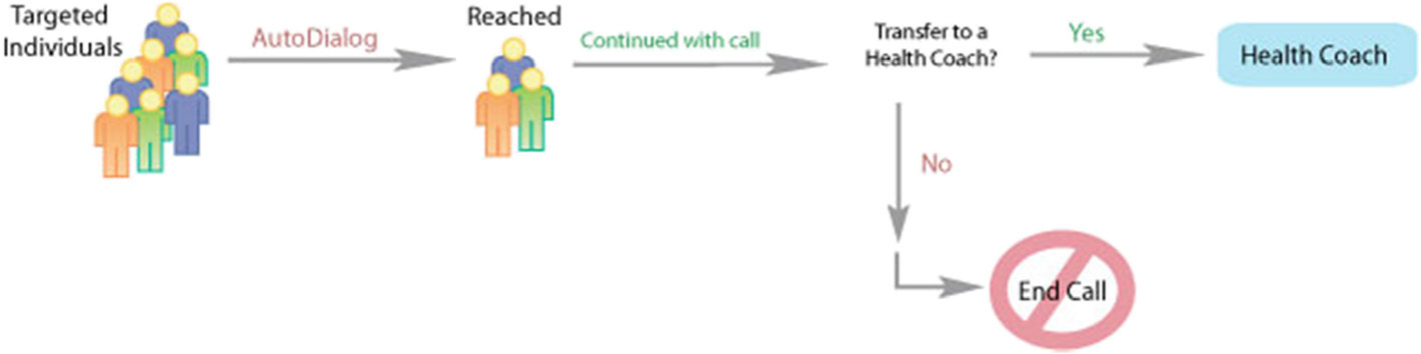
AutoDialog® Process.

The IVR system produced computer-generated outbound calls to individuals with health risks or conditions. The IVR technology simulated a one-on-one conversation and used a human rather than computerized voice. Individuals interacted with the technology using only their voice; touch-tone responses were not necessary as the technology recognized natural speech and interpreted the individual’s responses. Depending on his/her responses, the IVR system continued through a scripted call pathway. One pathway culminated in an offer to directly transfer the individual to a health coach. If the individual accepted the offer, the call automatically transferred to an available health coach. The health coach heard a “whisper” before the individual was connected, indicating the reason for the transfer (Figure 1).

All subjects studied (including subjects in the control group) were eligible to call in to the health coaching line. The key difference of the intervention was that the IVR calls® were expected to increase the number of targeted subjects speaking with health coaches.

### Control

For wave one, the control group received no mailed outreach. For waves two, three and four, the control group received mailed postcards. Because there were only very small differences in the health coaching rates in the control populations in the wave one and the other waves, we have not distinguished these two control group approaches in our analysis. The delivery of the postcards was aligned with the timing of the AutoDialog® calls scheduled for the intervention groups. The postcards contained messages that integrated the principles of the overall programs we were running with each health plan with a call-to-action to contact a health coach at a provided toll-free number. Collateral materials were modified and approved in collaboration with each program sponsor for branding consistence.

### Health coaching

The health coaching process did not differ for the intervention and control groups. Individuals selecting to participate with a health coach were provided personalized support. To the extent possible given the individual caller’s needs and interests, health coaches were expected to discuss the specific conditions and decisions related to those conditions for which the caller was targeted. The objective for health coaches when engaging individuals around preference-sensitive conditions was to tailor support so that person might become active participants in their treatment plans and engage in shared decisions with their health care providers. Health coaches were trained to meet quality and other performance standards for all calls in the study regardless of whether they resulted from the intervention or the control outreach. See Additional file 1: Appendix A for more details on the health coaching process.

### Study measures

The primary objective of these quality improvement studies was to assess the ability of IVR outreach to increase rates of health coaching among individuals with elevated risk of surgery for elective back and knee surgery compared to rates resulting from the control outreach. The denominators for all measures are defined on an intent-to-treat basis, meaning they include all subjects assigned to a given study arm except for those subjects meeting pre-defined exclusion criteria. All measures were assessed for the 6 month period post-outreach.

For all measures, month is defined as a 30-day interval, with the first interval starting the first day of the intervention for each wave. A subject is considered to have eligibility in a full 30-day interval if program records indicated eligibility in any calendar month within the 30 days. This assumption is required given the calendar month assignment of health plan eligibility.

### Health coach communication

The primary measure for the study is the rate of health coaching calls generated by the outreach. This is defined as the proportion of subjects in the study population with a health coach communication recorded in the health coaching application as an inbound call within thirty days of the start of the study. Calls to health coaches that connect through the IVR and calls that connect directly through the program toll-free number are both classified as inbound calls. Outbound calls were not included in the numerator for this measure. Outbound calls are calls health coaches place, either from lists generated through the outreach process or as follow-up calls scheduled during previous calls. Because of the randomization process, outbound call rates were not expected to be different for the control and intervention groups. In order to meet business objectives of the health plans, individuals in the study were not excluded from subsequent outbound call campaigns within the six month analysis window.

Secondary measures of communication include the total number of communications within the six months of the measurement period and the rate of video-based or booklet decision aid distribution during the measurement period. Decision aids were only sent to individuals who spoke with a health coach when the health coach assessed the need for a decision aid and the individual member agreed to receive the material. The criteria for health coach assessment of the need included, for decision aids related to herniated disc, knee osteoarthritis, and hip osteoarthritis, confirmation from the individual of a diagnosis from a health care provider.

### Surgical rates

Preference-sensitive back, hip, and joint surgeries for the entire study population were determined by assessing medical health care claims for six months following the intervention period. Medical claims captured details regarding ICD-9 diagnosis and CPT-4 procedure codes for back and joint surgeries (Additional file 2: Appendix B). The outcome is defined as any instance of one of the surgeries post-intervention. Joint surgery rates were analyzed separately from back surgery rates.

### Health care costs

We derived health care costs from paid medical claims data provided to Health Dialog by its clients; health plan clients provide these data under business associate agreements to enable Health Dialog to execute and evaluate a range of care management services. Costs were assessed by reviewing medical claims incurred for six months after the start of the respective study waves. The analysis focused on health plan paid amounts on the claims. Individuals were included in this analysis if they had at least one month of eligibility in the post-intervention period. In addition to total paid amounts, we also analyzed total paid amounts less costs associated with conditions and treatments that are both extremely unlikely to be affected by care management services and are sizable enough to potentially confuse interpretation of the results. We call the resulting costs “actionable costs”; actionable costs are total costs less costs associated with medical claims for trauma and accident, substance abuse, malignant neoplasm, and maternity and childbirth. All costs were converted to per member per month (PMPM) rates by dividing total expenses for the individual by the number of months the individual was eligible for Health Dialog’s services.

### Statistical analysis

We reported population characteristics as means and percentages. We used t-tests to determine similarities between the intervention and control groups at baseline for continuous variables (these tests were log-transformed if the data were not normally distributed). We used chi-square tests to assess characteristics expressed as percentages. Analysis of covariance was used to account for random imbalances in a set of parsimonious factors likely to be associated with measurement period costs between the AutoDialog® and the control group (age, gender, study wave, targeted preference-sensitive condition (overall model only), prior year’s facility, physician, ancillary, pharmacy costs, Health Coach communication in the prior year, continuous eligibility for the study period.) [17-20]. Logistic and Zero Inflated Poisson regression were used similarly for the dichotomous and count outcome measures. Model specifications are described in the result tables. All statistical analyses were performed with SAS software, version 9.2.

## Results

In total, the study included 24,167 individuals, 13,901 of whom were in the intervention group and 10,267 of whom were in the control group. Baseline differences between intervention and control groups on demographic variables and overall costs were not statistically significant within any wave (Table 3).

**Table 3:** Baseline characteristics by wave

	**Wave 1**		**Wave 2**		**Wave 3**		**Wave 4**	
**Characteristic**	**Intervention Group**	**Control Group**	**Intervention Group**	**Control Group**	**Intervention Group**	**Control Group**	**Intervention Group**	**Control Group**
Demographic Characteristics								
Total Population--N	4150	1366	5122	6430	3257	1074	1372	1396
Gender: Female--N (%)	2,355(56.8)	780(57.1)	3,342(65.3)	4,260(66.3)	2,020(62.0)	650(60.5)	806(58.8)	836(59.9)
Plan Type: Medicare Advantage--N(%)	0(0.0)	0(0.0)	2,131(41.6)	2,691(41.9)	1,426(43.8)	455(42.4)	0(0.0)	0(0.0)
Mean age--years	49.2	49	64.5	64.7	64.6	64.2	50.2	50.6
Age Group--N(%)								
0-30	157(3.8)	76(5.6)	24(0.5)	26(0.4)	29(0.9)	11(1.0)	45(3.3)	50(3.6)
30-39	581(14.0)	163(11.9)	97(1.9)	132(2.1)	119(3.7)	49(4.6)	160(11.7)	136(9.7)
40-49	1,229(29.6)	405(29.7)	533(10.4)	622(9.7)	363(11.2)	122(11.4)	400(29.2)	391(28.0)
50-59	1,528(36.8)	511(37.4)	1,292(25.2)	1,645(25.6)	718(22.0)	239(22.3)	524(38.2)	546(39.1)
60-64	541(13.0)	175(12.8)	732(14.3)	888(13.8)	453(13.9)	145(13.5)	172(12.5)	206(14.8)
65-74	100(2.4)	34(2.5)	1,134(22.1)	1,408(21.9)	616(18.9)	200(18.6)	62(4.5)	59(4.2)
75-84	14(0.3)	2(0.2)	964(18.8)	1,310(20.4)	714(21.9)	213(19.8)	8(0.6)	7(0.5)
85+	0(0.0)	0(0.0)	346(6.8)	399(6.2)	245(7.5)	95(8.9)	1(0.1)	1(0.1)
Prior Year Costs--$/person/month								
Total	469.55	420.29	578.13	568.09	471.06	512.77	505.89	601.95
Actionable	429.92	386.31	527.31	521.35	418.8	438.65	451.76	531.34
Physician	54.76	49.94	39.92	38.93	16.04	16.32	60.98	66.11
Facility	154.46	129.49	283.93	276.75	316.71	344.88	175.74	236.45
Pharmacy	80.12	70.91	66.92	65.4	79.28	76.33	72.8	80.05
Ancillary	180.2	169.96	187.37	187.07	59.03	75.24	197.95	220.4
Type of risk - N(%)								
Back	2,332(56.2)	750(54.9)	892(17.4)	1,088(16.9)	1,392(42.7)	515(48.0)	671(48.9)	682(48.9)
Joint	1,818(43.8)	616(45.1)	4,230(82.6)	5,342(83.1)	1,866(57.3)	559(52.1)	701(51.1)	714(51.2)
Prior Year Health Coaching -- N(%)	478(11.5)	142(10.4)	562(11.0)	663(10.3)	261(8.0)	78(7.3)	82(6.0)	92(6.6)

### Health coaching results

Individuals in the intervention groups were more likely than the control groups to make an inbound call within 30 days to a health coach; 11.1 percent of individuals in the intervention groups and 1.2 percent of individuals in the control groups called inbound (Table 4). In the 180 day period after the initial intervention, the intervention arm had 272.4 communications per 1,000 members and the control group had 125.5 communications per 1,000 member [modeled difference of 66 percent] (Table 5). These communications included outbound calls that resulted from ongoing campaigns outside of this study. These differences, along with differences on all the intent-to-treat health coaching communication measures, were statistically significant, even when controlling for such factors as historic utilization, gender, age and previous health coaching.

**Table 4:** 30 Day health coach contact rates per 1,000 members

	**Intervention arm**	**Control arm**	**Unadjusted differences**		**Odds ratio point difference**^**‡**^	**P value**
	**n = 13,901**	**n = 10,266**	**Count**	**Percent**	**(95% Wald CI)**	
Overall	111.09	12.2	98.89	810.6%	10.653(8.835 to 12.844)	<.0001
	n = 5,286	n = 3,035				
Preference-Sensitive Back Surgery	98.8	11.5	87.3	759.1%	10.173(7.161 to 14.451)	<.0001
	n = 8,615	7,231				
Preference-Sensitive Joint Surgery	118.3	12.4	105.9	854.0%	10.905(8.740 to 13.608)	<.0001

‡Modeled with logistic regression. Odds ratio adjusted for age category, gender, study wave, preference-sensitive condition (Overall model only), prior health coaching, prior year’s facility, physician, pharmacy and ancillary costs.

**Table 5:** 180 Days all health coach communications per 1,000 members

	**Intervention arm**	**Control arm**	**Unadjusted differences**		**Odds ration modeled difference**^**‡**^	**P value**
	**n = 13,901**	**n = 10,266**	**Count**	**Percent**	**(95% Wald CI)**	
Overall	272.42	125.46	146.96	117.1%	66.97%(58.78% to 75.15%)	<.0001
	n = 5,286	n = 3,035				
Preference-Sensitive Back Surgery	238.17	130.8	107.37	82.1%	45.26%(30.28% to 60.24%)	<.0001
	n = 8,615	n = 7,231				
Preference-Sensitive Joint Surgery	293.44	123.21	170.23	138.2%	75.59%(65.76% to 85.43%)	<.0001

‡Zero inflated Poisson regression - using number of Communications in prior 12 months as Zero inflation factor. Odds ratio adjusted for age category, gender, study wave, preference-sensitive condition (overall model only), prior year’s facility, physician, pharmacy and ancillary costs.

Individuals in the intervention group received 42.7 decision aids per 1,000 members, individuals in the control group received 16.1 decision aids per 1,000 members (Table 6). This was entirely a function of the higher contact rate for AutoDialog®. There were not differences between intervention and control in the proportion of contacted members who were sent decision aids; 33 percent of subjects contacted in both groups were sent decision aids within 30 days of contact.

**Table 6:** DVD decision aid distribution per 1,000 members

	**Intervention arm**	**Control arm**	**Unadjusted differences**		**Odds ratio point difference**^**‡**^	**P value**
	**n = 13,901**	**n = 10,266**	**Count**	**Percent**	**(95% Wald CI)**	
Overall	42.7	16.1	26.6	165.2%	3.154(2.635 to 12.844)	<.0001
	n = 5,286	n = 3,035				
Preference-Sensitive Back Surgery	43.1	20.1	23	114.4%	2.764(2.054 to 3.720)	<.0001
	n = 8,615	n = 7,231				
Preference-Sensitive Joint Surgery	42.4	14.4	28	194.4%	3.399(2.712 to 4.261)	<.0001

‡Odds ratio adjusted for age category, gender, study wave, preference-sensitive condition (overall model only), prior health coaching, prior year’s facility, physician, pharmacy and ancillary costs.

### Surgical rates results

The preference-sensitive surgery rate per 1,000 members was 34.3 in the intervention group and 38.9 in the control group. This was not a statistically significant difference after adjustment for appropriate potential covariates (Table 7).

**Table 7:** Preference-sensitive surgery rates per 1,000 members

	**Intervention arm**	**Control arm**	**Unadjusted differences**		**Odds ratio difference**^**‡**^	**P value**
	**n = 13,901**	**n = 10,266**	**Count**	**Percent**	**(95% Wald CI)**	
Any Preference-Sensitive Surgery	34.3	38.9	−4.6	−11.8%	0.963(0.834 to 1.111)	0.6039
	n = 5,286	n = 3,035				
Preference-Sensitive Back Surgery	18.7	21.1	−2.4	−11.4%	0.855(0.611 to 1.196)	0.3595
	n = 8,615	n = 7,231				
Preference-Sensitive Joint Surgery	36.6	40.2	−3.6	−9.0%	0.995(0.839 to 1.180)	0.9571

‡Odds ratio adjusted for age category, gender, study wave, preference-sensitive condition, continuous health plan eligibility, prior year’s actionable physician, professional and pharmacy costs within Logistic Regression with Preference-Sensitive Surgery as outcome.

### Health care costs results

Overall unadjusted per member per month costs in the 6 months after outreach were $595.88 in the intervention group and $637.54 in the control group [percentage difference was 6.5% unadjusted and 4.85% adjusted (p = 0.055)] (Table 8). This resulted in a mean cost difference of $41.67 PMPM. Unadjusted actionable per member per month costs (which exclude trauma, oncology and maternity) in the 6 months after outreach were $479.60 in the intervention group and $524.88 in the control group [percentage difference was 10.3% unadjusted and 6.45% adjusted (p = 0.029)]. There was also a statistically significant difference in actionable costs within the group with joint surgery risks. The actionable cost differences for individuals with back surgery risks were not statistically significant. The intervention group had consistently lower costs than the control group in all service categories except pharmacy, where costs were higher.

**Table 8:** Per member per month costs

	**Intervention arm**	**Control arm**	**Unadjusted differences**		**Modeled percent difference**^**‡**^	**P value**
			**Cost**	**Percent**	**(95% CI)**	
**All Members**	n = 13,901	n = 10,266				
All PMPM Costs	$595.88	$637.54	-$41.67	−6.5%	−0.0485(−0.0981 to 0.0011)	0.0555
†Actionable PMPM Costs	$470.60	$524.88	-$54.28	−10.3%	−0.0645(−0.1222 to −0.0067)	0.0286
All PMPM Physician Costs	$37.66	$40.03	-$2.38	−5.9%	−0.0389(−0.0769 to -.0008)	0.0451
All PMPM Facility Costs	$321.51	$344.57	-$23.07	−6.7%	−0.0568(−0.1259 to 0.0122)	0.1066
All PMPM Pharmacy Costs	$80.42	$71.66	$8.76	12.2%	−0.0070(−0.0376 to 0.0236)	0.6524
All PMPM Ancillary Costs	$156.29	$181.28	-$24.99	−13.8%	−0.0396(−0.0923 to 0.0130)	0.1401
**Members with Risks for Preference-Sensitive Back Surgery**	n = 5,286	n = 3,035				
ll PMPM Costs	$534.06	$589.32	-$55.26	−9.4%	−0.0391(−0.1232 to 0.0450)	0.3617
†Actionable PMPM Costs	$399.94	$458.68	-$58.73	−12.8%	−0.0342(−0.1337 to 0.0652)	0.4999
All PMPM Physician Costs	$39.87	$41.40	-$1.53	−3.7%	−0.0153(−0.0820 to 0.0514)	0.6533
All PMPM Facility Costs	$261.85	$296.09	-$34.24	−11.6%	−0.0150(−0.1305 to 0.1005)	0.7989
All PMPM Pharmacy Costs	$92.08	$86.92	$5.16	5.9%	−0.0428(−0.0986 to 0.0131)	0.1338
All PMPM Ancillary Costs	$140.26	$164.92	-$24.65	−14.9%	−0.0393(−0.1298 to 0.0512)	0.3943
**Preference-Sensitive Joint Surgery**	n = 8,615	n = 7,231				
All PMPM Costs	$633.81	$657.79	-$23.98	−3.6%	−0.0523(−0.1138 to 0.0091)	0.0948
†Actionable PMPM Costs	$513.96	$552.67	-$38.71	−7.0%	−0.0747(−0.1456 to −0.0039)	0.0387
All PMPM Physician Costs	$36.30	$39.46	-$3.16	−8.0%	−0.0497(−0.0961 to −0.0034)	0.0353
All PMPM Facility Costs	$358.11	$364.92	-$6.81	−1.9%	−0.0720(−.1580 to 0.0139)	0.1005

† Excludes claims associated with trauma & accident, psych/substance abuse, malignant neoplasm, maternity and childbirth. Excludes patients with no health plan eligibility in the pre or post period.

‡Adjusted for age, gender, study wave, targeted preference-sensitive condition (overall model only), prior year’s facility, physician, ancillary, pharmacy costs, Health Coach communication in the prior year, continuous eligibility for the study period.

## Discussion

Across all four waves, subjects targeted by AutoDialog® were much more likely to connect with a health coach compared to subjects in the control groups. This finding alone supports the active ongoing use of AutoDialog® and similar programs to encourage shared-decision making coaching (as well as coaching for other needs and risks).

The strong association between this increased level of health coaching and statistically significant reductions in actionable medical costs is confirming of the importance of and potential impact of health coaching for people with specific surgical risks. Health coach support, for subjects at key decision points, can enhance the ability of individuals to manage their own conditions and make informed decisions. The results of these studies, finding statistically significant reductions of 6.45 percent on actionable costs, provide powerful evidence that telephonic delivery of health coaching to a population without five common chronic conditions (asthma, chronic obstructive pulmonary disease, coronary heart disease, diabetes, and heart failure) who are identified at risk for back and hip or knee repair and replacement preference-sensitive surgery lowers medical costs. This finding is supported by a subsequent, large scale trial of Health Dialog’s care management program, which prominently featured both the use of IVR and targeting of individuals at elevated risk for preference-sensitive surgeries, found significant savings for a group in which more individual participated in health coaching and were coached more intensely compared to less health coaching [21].

The lack of statistically significant impact on surgery rates raises several important issues. First, given the relatively low rate of surgery in the control group, significant impact on surgery rates was difficult to detect. Second, the cost reduction described is not explainable simply by the measured changes in surgery rates. This cost reduction may have resulted from improvements in general self-care and navigation skills or may have resulted from knowledge and decision making that impacted much more than the decisions about surgeries.

While other mechanisms for engaging individuals to support their health care decisions exist, including provider-based, employer work-site based, electronic (e.g., web-based), and mailed education and support processes, direct telephonic communication with a health coach appears to provide important substantive support at the same time as being more easily distributed than on-site support. The results of these studies support the ongoing use of health coaching to improve health care utilization and reduce medical expense. Providing support for people with specific risks for surgery without common chronic conditions appears to be a beneficial focus.

### Limitations

There are four limitations worth considering when interpreting these findings. First, the vast majority of subjects, even in the treatment arm, never received any health coaching or shared decision making materials. The opportunity for such support was available to all, but the most did not take advantage of it.

Secondly, the surgical prevalence rates were under 5%. This study was never reasonably powered for surgeries, which brings into question the source of the savings. While detailed analysis of the actual mechanism of the cost savings is infeasible due to power issues, it is noteworthy that the AutoDialog® group has lower ancillary costs, which includes expensive imaging procedures which tend to occur as part of the clinical pathway leading to surgery. The significant savings found for actionable costs seems to be the results of lower facility costs and lower ancillary costs in the group receiving more coaching and shared decision making support.

Third, the distribution level of decision aids is low. No more than 4.3% of subjects targeted were sent a video decision aid. This is related primarily to the low surgical rates, which indicate that a low percentage of subjects reasonably needed a surgical decision aid. At the same time, the sparseness of distribution raised questions regarding the independent impact of decision aids and shared-decision making informed health coaching. Subsequent research conducted by Health Dialog has examined this issue. In a study of pervasive distribution of a Heart Failure video, recipients of a mailed Heart Failure DVD decision aid reported significantly higher levels of daily weight monitoring [22].

Finally, although the health coaches who were in this study were trained to treat all participants similarly without regard whether they were in the intervention or control group, the health coaches had information about the name of the campaign that each person was associated with. It was infeasible to blind the health coaches to the study. These two facts (the person-level information about the campaign and the overall exposure to the study) pose the risk of bias in the health coaching that was provided.

## Conclusions

Providing high rates of health coaching to individuals with risks of lumbar back, hip replacement, or knee replacement surgery can appropriately reduce health costs. The ability of health coaching to moderate health cost increases for other health conditions was not studied, but there may be value in assessing whether tailored health coaching can impact health costs for individuals with other conditions. Interactive voice response phone calls that enable direct transfer to health coaches are highly effective at efficiently increasing health coaching rates.

## Abbreviations

CPT-4: Current Procedural Terminology, 4th Edition; DVD: Digital video disc; ICD-9: International Statistical Classification of Diseases and Related Health Problems (9^th^ revision); PMPM: Per member per month.

## Competing interests

Mr. Veroff is a current employee and Ms. Ochoa-Arvelo and Mr. Venator are past employees of Health Dialog. Health Dialog financed the costs of the research and publication. Mr. Veroff and Mr. Venator formerly held stock options in Health Dialog, but all options have been exercised.

## Authors’ contributions

All authors contributed substantially to this research including (1) the conception and design of the study (DRV, BV), (2) the execution of the tested programs (DRV, BV), (3) analysis and interpretation of data (all authors), (4) drafting the article or revising it critically for important intellectual content (all authors), (5) final approval of the version to be submitted (all authors). The corresponding author, DRV, is responsible for the integrity of the work as a whole.

## Pre-publication history

The pre-publication history for this paper can be accessed here:

http://www.biomedcentral.com/1472-6947/13/21/prepub

## Supplementary Material

Additional file 1: Appendix A Health Coaching. Click here for file

Additional file 2: Appendix B Preferences-Sensitive Surgical Codes. Click here for file
