# Supplementary material for: A randomized study of telephonic care support in populations at risk for musculoskeletal preference-sensitive surgeries
Source: BMC Med Inform Decis Mak. 2013 Feb 7;13:21. doi: 10.1186/1472-6947-13-21 (PMC3575312; doi:10.1186/1472-6947-13-21)
Supplement: Additional file 1: Appendix A — Health Coaching. [file 1472-6947-13-21-S1.doc]

**Appendix A: Health Coaching**

Health coaching was delivered telephonically from licensed clinical staff (registered nurses, dieticians, respiratory therapists, and pharmacists) to individual health plan members and other health care consumers.

Health coaches were guided and supported in their interactions with members by a customized software application that integrates reference material and guides for discussion, imbeds logical prioritization of clinical concerns based on rich analytics, and enables direct access to clinical facts derived from medical and pharmacy claims, survey responses, clinical data, and prior health coaching interactions. Health coaches used this same software to record the content of interactions with members. Health coaches were also instructed to support people in the process of making major health care decisions using Health Dialog’s proprietary Shared Decision Guide which guided the health coaches on the topics and material to cover to support shared decision making.

Health coaches sent mailings, email, and web links to study participants from their educational health materials list, which included 17 decision support videos and 10 self-management videos, 15 self-monitoring aids (response and medication planners, scales, diaries, foot care kits), 110 booklets and pamphlets, and an interactive health library (Healthwise® Knowledgebase) that allowed health coaches to create customized information packets on approximately 8,000 medical topics.
