# Supplementary figures and images for: A randomized study of telephonic care support in populations at risk for musculoskeletal preference-sensitive surgeries
Source: BMC Med Inform Decis Mak. 2013 Feb 7;13:21. doi: 10.1186/1472-6947-13-21 (PMC3575312; doi:10.1186/1472-6947-13-21)

**Appendix B: Preference-Sensitive Surgical Codes**


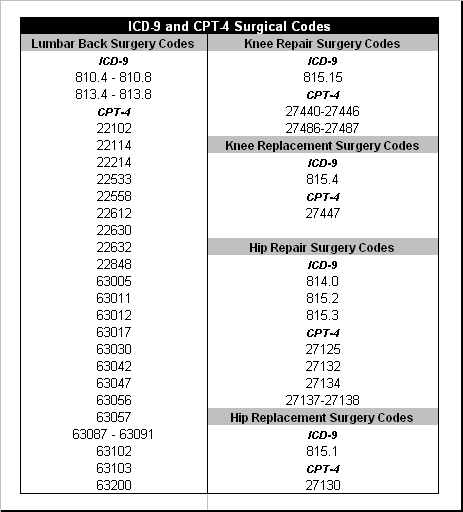

Supplement: Additional file 2: Appendix B — Preferences-Sensitive Surgical Codes. [file 1472-6947-13-21-S2.doc]
